# A proposed psychometabolic endotype of polyendocrine metabolic ovarian syndrome: bidirectional interplay between psychological distress, eating dysfunction, sleep disruption, chronic stress, and metabolic deterioration

**DOI:** 10.3389/fendo.2026.1978249

**Published:** 2026-10-07

**Authors:** Hongtao Li, Tong Yin, Yichen Wang, Yixuan Wang, Nenggui Xu, Wei Yi, Han Yang

**Affiliations:** 1 South China Research Center for Acupuncture and Moxibustion, School of Acupuncture-Moxibustion and Rehabilitation, Guangzhou University of Chinese Medicine, Guangzhou, China; 2 The Second Clinical Medical College, Guangzhou University of Chinese Medicine, Guangzhou, China; 3 Tianjin University of Traditional Chinese Medicine, Tianjin, China; 4 School of Nursing, Guangzhou University of Chinese Medicine, Guangzhou, China

**Keywords:** anxiety, depression, endotype, insulin resistance, polycystic ovary syndrome, polyendocrine metabolic ovarian syndrome, precision medicine

## Abstract

**Background:**

Polycystic ovary syndrome was renamed polyendocrine metabolic ovarian syndrome (PMOS) in 2026 to better represent its multisystem biology. Psychological distress, eating dysregulation, sleep disturbance, and metabolic dysfunction frequently coexist, yet clinical care and research commonly treat them as parallel comorbidities.

**Objective:**

To define the proposed psychometabolic construct operationally; position it relative to conventional phenotypes and data-driven clusters; and grade evidence for association, temporal prediction, causation, and treatment response across psychological, behavioral, sleep–stress, neuroendocrine, and metabolic pathways.

**Methods:**

We conducted a targeted narrative review of PubMed/MEDLINE from inception through 15 September 2026. Seven structured topic blocks returned 1,278 records, 1,009 unique identifiers after PubMed identifier (PMID)-based deduplication, and 1,008 retrievable article records for title/abstract screening. Guidelines, systematic reviews, longitudinal or causal studies, trials, and informative human mechanistic studies were prioritized; general-population and preclinical evidence was used only when its indirectness was explicit.

**Evidence synthesis:**

Depression and anxiety, body-image concerns, binge- and bulimia-spectrum symptoms, poor sleep, and obstructive sleep apnea are reported more often in PMOS than in comparison populations, but most estimates are cross-sectional and heterogeneous. One tertiary cohort found that baseline depression and/or anxiety history predicted incident metabolic syndrome over a median seven years (adjusted hazard ratio 1.45, 95% confidence interval 1.02–2.06); this supports prognostic enrichment, not causality. General-population longitudinal and genetic studies support reciprocal depression–metabolic relationships, whereas PMOS-specific causal evidence is inconsistent. Plausible links include insulin–androgen–adipose interactions, neuroactive steroid signaling, ovarian sympathetic input, sleep fragmentation, stress and autonomic regulation, reward-related eating, treatment exposure, stigma, and social context. None validates a discrete endotype.

**Conclusions:**

We define a candidate psychometabolic endotype as a cross-cutting, mechanistically coupled pattern that may occur within any conventional PMOS phenotype. Clinical co-occurrence is only an entry profile; endotype status requires replicated longitudinal coupling, incremental prognostic value, interpretable transitions, and preferably treatment-effect heterogeneity. Four overlapping prototypes are working formulations rather than subtypes. A six-step clinical pathway centers shared formulation and nests care-intensity tiers within treatment selection.

## 1 Why PMOS needs a psychometabolic framework

The 2026 international consensus renamed polycystic ovary syndrome (PCOS) as polyendocrine metabolic ovarian syndrome (PMOS), shifting emphasis toward interacting endocrine, metabolic, reproductive, and life-course manifestations without immediately altering diagnostic criteria (1). We use PMOS for the historical condition and retain PCOS in source titles, instruments, and diagnostic frameworks. The 2023 guideline remains the operative evidence base (2–6), and contemporary syntheses document marked clinical heterogeneity (7–12).

The rename creates an opportunity—but not proof—for integrated mental and metabolic care. Depression may disrupt sleep and self-care; restriction may precipitate loss-of-control eating; stigma may delay care; sleep fragmentation may worsen appetite and insulin sensitivity; and metabolic or androgenic manifestations may reinforce distress. Treatment benefit, adverse effects, burden, cost, and withdrawal can interrupt or amplify these loops, which may converge in some people and remain independent in others.

Core domains—depression/anxiety, body image/stigma, eating pathology, sleep/obstructive sleep apnea (OSA), and stress–autonomic regulation—were selected for the targeted narrative synthesis because they recur in PMOS guidance, plausibly affect behavior or metabolic physiology, and offer measurable, modifiable targets (2, 13, 14). Bipolar, psychotic, attention-deficit/hyperactivity, and autism spectrum conditions were not core domains because PMOS-specific longitudinal and mechanistic evidence is less developed or heterogeneous; they remain important comorbidities, differential diagnoses, and treatment-exposure contexts.

### 1.1 Review scope, search strategy, and study selection

The narrative synthesis was supported by seven structured PubMed/MEDLINE searches conducted during revision to improve the transparency and reproducibility of source identification. The searches covered mental health/lived experience, eating, sleep/circadian function, stress–autonomic–neuroendocrine mechanisms, longitudinal/causal evidence, treatment, and phenotypes/endotypes/clusters. Searches covered database inception through 15 September 2026. Exact strings and yields appear in **Additional File 1**. They returned 1,278 records, 1,009 unique PubMed identifiers after deduplication, and 1,008 retrievable article records for title/abstract screening. All record counts reflect searches executed on 15 September 2026.

The authors conducted a single-stage title/abstract relevance screen of the 1,008 retrievable records. Potentially informative reports were subsequently checked in full text for population, domain, study design, evidential role, and directness to PMOS. English-language sources were eligible if they addressed PMOS/PCOS and a selected domain, mechanism, temporal relation, assessment, or treatment. Guidelines, systematic syntheses, longitudinal/causal studies, trials, and human mechanistic studies were prioritized. Cross-sectional evidence informed burden; preclinical evidence was used sparingly for biological plausibility; and general-population evidence was included only to address gaps and was labeled indirect. Unretrievable reports, isolated case reports, and unrelated studies were excluded. Reference chaining and society or publisher records supplemented PubMed.

Selection followed evidential role: association, temporality, causal inference, intervention, or implementation. Overlapping reviews were resolved by recency and methodological informativeness. Because this was a targeted narrative review, we did not recalculate pooled estimates, apply a formal risk-of-bias tool, maintain an exhaustive full-text exclusion log, or claim a PRISMA-compliant systematic-review workflow. Limitations and indirectness accompany each claim.

## 2 Defining a candidate psychometabolic endotype

### 2.1 Terminology and operational definition

A phenotype is an observable pattern; a symptom cluster comprises co-varying complaints; a clinical subtype is a pragmatic grouping; and a comorbidity is a separate condition occurring with PMOS. An endotype is narrower: a subgroup distinguished by a reproducible mechanism with prognostic or treatment relevance. Thus, PMOS with depression and obesity is a comorbidity pattern unless linked mechanisms add information beyond the components.

Operational definition. The candidate psychometabolic endotype is a cross-cutting (transversal) mechanistic construct that may occur within any conventional PMOS phenotype or data-driven cluster. It is not a fifth National Institutes of Health (NIH)/Rotterdam phenotype, not itself an unsupervised cluster, not an established PMOS subtype, and not a name for each individual mechanism.

A clinical entry profile requires at least one psychological, eating, or sleep–stress feature plus at least one metabolic or reproductive feature. Co-occurrence triggers joint assessment but does not establish an endotype.

Validation requires (1) reproducible cross-domain coupling after appropriate control of common causes and treatment exposure (2); temporal stability or interpretable transitions on repeated measurement (3); incremental prognostic value beyond conventional phenotype, adiposity, medications, and social context; and ideally (4) replicated treatment-effect heterogeneity from a mechanism-matched intervention.

Accordingly, we propose one cross-cutting framework. The four prototypes are overlapping entry patterns—not separate endotypes or fixed categories—and insulin resistance, neuroactive steroids, autonomic activity, eating, and sleep are component mechanisms. These distinctions and the status of the proposed construct are summarized (**Table 1**).

**Table 1:** Terminology and the status of the proposed construct.

Term	What it denotes	Relationship to the candidate construct
NIH/Rotterdam phenotype	Observable reproductive–endocrine feature combination	Conventional phenotype remains primary; candidate coupling may occur within any phenotype.
Symptom cluster	Co-varying symptoms without a demonstrated mechanism	May trigger assessment but is insufficient for endotype status.
Data-driven cluster	Empirical grouping generated by an algorithm	May overlap the construct; stability and mechanism still require validation.
Candidate mechanism	A proposed link such as neuroactive steroid or autonomic signaling	One component of the network, not an endotype by itself.
Working prototype	Overlapping clinical entry pattern used for formulation	Not a subtype, diagnosis, or validated endotype.
Candidate psychometabolic endotype	Cross-cutting mechanistic coupling with replicated prognostic or treatment value	Research construct; no current diagnostic assignment.

### 2.2 Relationship to NIH/Rotterdam phenotypes and data-driven clusters

Classic phenotypes remain descriptive. The National Institutes of Health (NIH) construct requires hyperandrogenism and ovulatory dysfunction after exclusion of alternatives. Rotterdam criteria require two of hyperandrogenism, ovulatory dysfunction, and polycystic ovarian morphology, yielding phenotypes A (all three), B (first two), C (hyperandrogenism plus morphology), and D (ovulatory dysfunction plus morphology) (3–6). None encodes a psychological–metabolic mechanism; the candidate pattern may be present or absent in any phenotype.

Unsupervised analyses identify reproductive, metabolic, intermediate, or background clusters from anthropometry, gonadotropins, and metabolic markers (15–19). Dapas et al. replicated reproductive/metabolic clusters and reported genetic signals, but ancestry, NIH case definition, small subtype analyses, and absent psychiatric measures constrain generalization (19). These studies support heterogeneity, not psychometabolic coupling; any future overlap still requires mechanistic validation.

### 2.3 Four overlapping working prototypes—not subtypes

Four overlapping, changeable prototypes support shared formulation and prospective testing:

• Distress-dominant: mood, infertility-related distress, body image, or stigma predominates. Prediction: repeated distress adds to metabolic prognosis.
• Eating-dysregulation: bingeing, loss of control, bulimia-spectrum symptoms, or severe restriction organizes the loop at any size. Prediction: symptom improvement precedes better adherence, glycemic variability, or weight stability.
• Sleep–stress–autonomic: OSA, insomnia, circadian disruption, hyperarousal, or fatigue predominates. Prediction: objective sleep improvement precedes emotional/metabolic improvement beyond weight change.
• Metabolic-treatment-sensitive: insulin resistance, dysglycemia, adiposity, or rapid weight change predominates and mental health varies with symptoms/treatment. Prediction: biological and psychological responses covary reproducibly.

These are not diagnoses or rationing categories; several may coexist or change with life stage and treatment.

### 2.4 A bidirectional multilevel model

Four domains interact: psychological distress, eating dysregulation, sleep–stress–autonomic regulation, and metabolic–reproductive biology. Lived context and treatment modify each. Bidirectional effects occur over different timescales, from nightly sleep change to multiyear dysglycemia (**Figure 1**).

**Figure 1:**
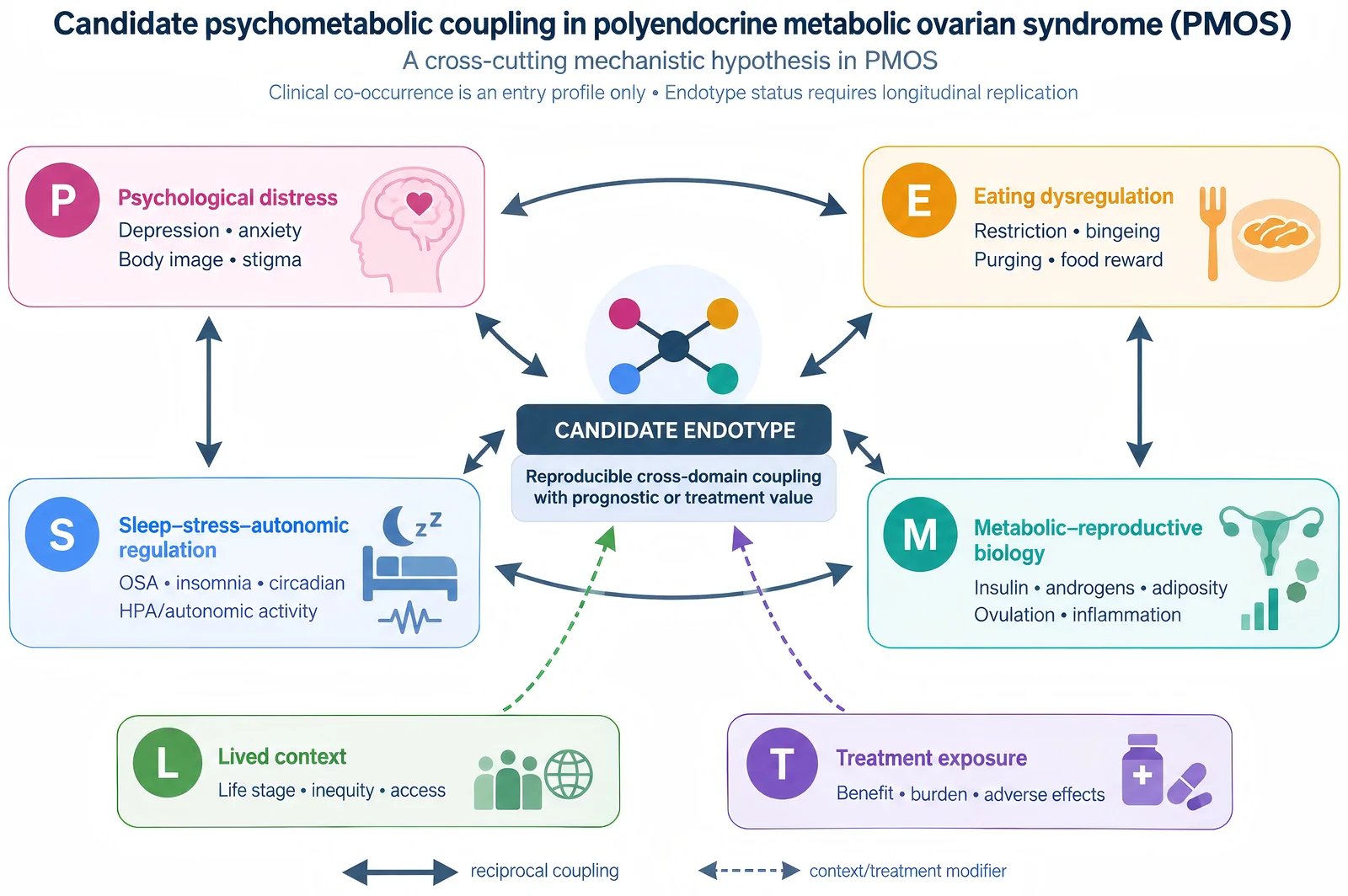
Candidate psychometabolic coupling in polyendocrine metabolic ovarian syndrome (PMOS). The proposed construct is a cross-cutting mechanistic hypothesis connecting psychological distress, eating dysregulation, sleep–stress–autonomic regulation, and metabolic–reproductive biology, with lived context and treatment exposure acting as modifiers. Solid bidirectional arrows denote hypothesized reciprocal coupling, whereas dashed arrows denote contextual or treatment modification. Clinical co-occurrence represents an entry profile only; endotype status requires replicated longitudinal coupling, incremental prognostic value, and preferably treatment-effect heterogeneity. Colors are reused across figures only when they denote the same concept. The schematic is an author-proposed research framework and not a diagnostic classifier. HPA, hypothalamic–pituitary–adrenal; OSA, obstructive sleep apnea; PMOS, polyendocrine metabolic ovarian syndrome.

The model avoids psychological reductionism, biological determinism, and treating body mass index (BMI) as the mechanism. Adiposity may be modifier, mediator, confounder, or outcome depending on the question; repeated measurement is therefore preferable to permanent type assignment.

## 3 Emotional health burden: robust association, incomplete explanation

### 3.1 Depression and anxiety

An overview of ten systematic reviews reported pooled prevalence estimates of 34.8% for depressive disorders and 16.9% for nonspecific anxiety disorders, while symptom prevalence varied widely by instrument (20). A meta-analysis of 30 cross-sectional studies found higher odds of any depressive symptoms (odds ratio [OR] 3.78, 95% confidence interval [CI] 3.03–4.72), moderate-to-severe depressive symptoms (OR 4.18, 2.68–6.52), any anxiety symptoms (OR 5.62, 3.22–9.80), and moderate-to-severe anxiety symptoms (OR 6.55, 2.87–14.93) than in controls (21). Associations persisted in a small subset of BMI-matched comparisons, but matching cannot eliminate residual confounding or determine direction. Other meta-analyses broadly support excess depression, anxiety, and psychiatric diagnoses while documenting heterogeneity in setting, case definition, comparator selection, and measurement (22–27).

Meta-analyses in adolescents and young adults also report more depression and anxiety and lower self-esteem, although studies are few and predominantly cross-sectional (28, 29). Postpartum depression may be more common after pregnancies affected by PMOS, but infertility treatment, obstetric complications, adiposity, prior depression, and ascertainment complicate inference (30). Guidelines therefore recommend routine assessment with attention to severity and safety (2, 13). Screening estimates risk; they do not establish a psychiatric diagnosis.

### 3.2 Body image, stigma, and diagnostic experience

A 2023 meta-analysis of nine studies found greater appearance- and weight-related concerns in PMOS but judged the evidence limited (31). Health-related quality of life varies across weight, infertility, menstrual, emotional, and hirsutism domains (32); condition-specific instruments such as the Polycystic Ovary Syndrome Questionnaire (PCOSQ) and PCOS Quality of Life Scale (PCOSQOL) make these burdens visible without establishing mechanism (33, 34). Individual and qualitative studies link symptom burden, body dissatisfaction, delayed diagnosis, inadequate information, dismissal, and fragmented care with distress (35–41).

Weight stigma is an exposure rather than a synonym for body size. Anticipated judgment may delay care, reduce disclosure of bingeing, and turn weighing or dietary counseling into a threat cue. A weight-inclusive approach separates metabolic risk assessment from moral judgment, asks permission before discussing weight, and avoids attributing fatigue, infertility, or distress to weight alone. This stance does not deny metabolic risk; it improves the conditions under which risk can be assessed and treated.

### 3.3 What longitudinal evidence adds

In the Northern Finland Birth Cohort, questionnaire-defined PMOS symptoms were associated with distress at ages 31 and 46; BMI and measured hyperandrogenism did not explain the pattern, while awareness of diagnosis was associated with anxiety (42). In a specialty cohort of 163 participants followed for a median 5.5 years, depressive-screen positivity was relatively stable and higher BMI predicted persistent risk, although the estimate was imprecise (43). An earlier follow-up retained only 60 of 103 participants and observed an estimated 19% conversion to depression over about 22 months (44). Selection, attrition, self-report, and small samples preclude causal claims but argue against assuming distress will resolve after diagnosis.

## 4 Eating behavior as outcome, mediator, and safety consideration

### 4.1 Prevalence and pattern

Eating pathology in PMOS includes loss-of-control eating, binge-eating disorder, bulimia nervosa, emotional or night eating, rigid restraint, chaotic timing, purging, and clinically significant shape or weight overvaluation. In the systematic review supporting the 2023 guideline, 20 studies included 28,922 participants with PMOS and 258,619 controls. PMOS was associated with any eating disorder (OR 1.53, 95% CI 1.29–1.82), bulimia nervosa, binge-eating disorder, and higher disordered-eating scores, including in weight-stratified analyses; anorexia nervosa was not increased (45). An earlier meta-analysis estimated ORs of 3.05 for an abnormal screen and 3.87 for any diagnosis, but with wide CIs (46). Other reviews converge on binge and emotional-eating patterns while emphasizing heterogeneity and weak longitudinal evidence (47–51).

Original studies report greater cravings and binge symptoms in subsets of adults and adolescents (52–55). Body size is an unreliable proxy because eating disorders occur across BMI categories. A 2026 adolescent cohort reported that disordered-eating behavior preceded later PMOS risk (56). This establishes temporal order for one direction but not causation; pubertal development, weight change, genetics, stress, and healthcare contact may influence both.

### 4.2 Restriction–binge cycles, food palatability, neuroimaging, and insulin dynamics

A clinically common hypothesis is that distress or stigma prompts rigid restriction; restriction increases hunger and food salience; loss-of-control eating produces shame; and shame drives renewed restriction and avoidance of care. Evidence from bulimia nervosa and related eating disorders makes parts of this loop biologically plausible but remains indirect to PMOS. An activation-likelihood meta-analysis of functional magnetic resonance imaging (fMRI) studies reported altered reward and interoceptive responses to food cues in bulimia nervosa, while a food-choice experiment implicated altered integration of healthiness and tastiness in ventromedial prefrontal valuation (57, 58). A systematic review found greater preference for sweetness in bulimia nervosa across several psychophysical studies, although methods and findings varied (59).

Experimental metabolic findings are mixed and argue against a simple insulin-resistance explanation. A small meal-and-purge study observed acute reductions in glucose and insulin after purging that could reinforce repeated episodes (60). Another study found attenuated postprandial glucagon-like peptide-1, pancreatic polypeptide, and insulin despite normal glucose (61). In contrast, a small intravenous insulin-tolerance study found no chronic insulin resistance in normal-weight participants with bulimia nervosa despite frequent polycystic ovarian morphology (62). These studies are small, old in part, and not PMOS-specific; they support investigation of acute glycemic flux, satiety signaling, and reward learning, not a claim that basal insulin resistance causes bulimia in PMOS.

The immediate clinical implication is safety. Prescribing an aggressive energy deficit without asking about bingeing, purging, compensatory exercise, or severe restriction may worsen the target process. A positive screen should prompt diagnostic assessment by an appropriately trained clinician, not exclusion from all metabolic care. Goals can shift toward regular nourishment, reduction of binge frequency, sleep stabilization, and cardiometabolic monitoring while specialist treatment proceeds.

## 5 Sleep and circadian disruption: modifiable but confounded

### 5.1 Sleep disturbance and obstructive sleep apnea

Poor sleep quality, short sleep, daytime sleepiness, insomnia, circadian misalignment, and OSA overlap but are not interchangeable. A 2025 meta-analysis of eight cross-sectional studies estimated OSA prevalence at 37% in PMOS versus 6% in non-PMOS comparators and a pooled OR of 9.52, with moderate heterogeneity and higher prevalence in adults, participants with higher BMI, and White populations (63). Other reviews report excess circadian disruption, OSA, and broadly defined sleep disturbance (64–67). A separate meta-analysis estimated 35% OSA prevalence but emphasized selection bias, predominantly United States samples with class II obesity, and inadequate confounder control (68).

Within PMOS, OSA is associated with adiposity, blood pressure, insulin resistance, dyslipidemia, and dysglycemia. However, a six-study synthesis noted that most studies did not adjust for weight and could not establish an independent effect (69). In a small, carefully phenotyped study, OSA prevalence was 56% in PMOS versus 19% in age- and BMI-similar controls (adjusted OR 7.1); OSA was associated with higher homeostatic model assessment of insulin resistance (HOMA-IR) within PMOS (70). Earlier physiological studies and an adolescent clamp study support concern but remain small (71–73). These data justify symptom-based case-finding, not universal attribution.

Snoring, witnessed apneas, nonrestorative sleep, morning headache, resistant hypertension, and excessive daytime sleepiness should prompt formal evaluation, especially when metabolic measures deteriorate unexpectedly. Standard scores may underperform in younger women because classic OSA phenotypes were developed in older or male-dominant populations. Diagnosis should follow local sleep guidance using polysomnography or appropriately validated home testing.

### 5.2 Circadian timing and metabolic behavior

Circadian disruption can alter meal timing, activity, cortisol rhythms, and sleep duration, while PMOS symptoms and treatments may disrupt schedules. PMOS-specific evidence is dominated by cross-sectional chronotype, melatonin, and sleep-quality studies (64). A population study of middle-aged women with previous PMOS links chronotype with health characteristics but cannot separate lifelong preference from work, caregiving, menopause, or current metabolic status (74). Regular sleep–wake and meal timing are low-risk targets; claims of a circadian endotype remain premature.

## 6 Stress biology and autonomic regulation

Stress operates at several levels: symptoms and infertility are stressors; stigma and diagnostic uncertainty shape appraisal; depression and anxiety alter perceived and physiological stress; and endocrine or autonomic systems may modify downstream metabolic responses. These levels should not be collapsed into a single cortisol explanation.

A laboratory study of 32 patients and 32 BMI-matched controls found greater adrenocorticotropic hormone (ACTH) and cortisol responses to public-speaking stress, higher heart rate, and altered interleukin-6 response in PMOS (75). A hair-cortisol study reported higher concentrations in 44 patients than in 49 controls and correlations between androgens and metabolic-inflammatory markers only in the high-cortisol subgroup (76). Both are compatible with hypothalamic–pituitary–adrenal (HPA) axis alteration; neither shows that chronic stress causes metabolic deterioration. Sampling, hormonal treatment, hair processing, assay differences, acute context, and unmeasured adversity can change estimates.

Autonomic studies provide a second signal. Meta-analyses report reduced time-domain heart-rate variability (HRV), a higher low-frequency/high-frequency ratio, and greater muscle sympathetic nerve activity (77, 78). Small microneurographic and physiological experiments support sympathoexcitation and possible modification by exercise or low-frequency electroacupuncture (79–83). HRV nevertheless depends on respiration, fitness, medication, sleep, posture, and analytic choices; samples are generally modest and measurements cross-sectional (84). These findings are mechanistic signals, not a clinical test for the proposed endotype.

Stress biology has a social distribution. Food insecurity, shift work, caregiving, discrimination, unsafe environments, and treatment cost may generate both distress and metabolic risk. Omitting them can biologize structural exposure; dismissing physiology as mere epiphenomenon may miss amplification. Context and biomarkers should be measured together with repeated sampling and prespecified mediation analyses.

## 7 Shared biological pathways: convergence without biomarker determinism

### 7.1 Insulin–androgen–adipose interactions

Insulin resistance and hyperinsulinemia affect many, but not all, people with PMOS. Insulin can increase ovarian androgen production and reduce sex hormone-binding globulin (SHBG); androgen excess and adiposity may worsen insulin action and fat distribution (85, 86). These links affect symptoms and fertility but do not reduce depression or eating pathology to endocrine symptoms: BMI, insulin resistance, and androgens explain only part of mental-health differences (21, 42).

Adipose expansion, ectopic lipid, adipokines, and immune signaling may connect weight trajectory to insulin and vascular risk. Meta-analyses report higher C-reactive protein (CRP) and other inflammatory/oxidative signals with heterogeneity by adiposity, assay, and quality (87–89). Adipokine findings also vary (90–93); no marker identifies a clinical psychometabolic endotype (94).

### 7.2 Neuroactive steroids and central regulation

Progesterone- and androgen-derived neuroactive steroids can modulate gamma-aminobutyric acid type A (GABA-A) receptors, linking steroid biology with affect, stress responsivity, inhibition, and reproductive feedback. In addition to the progesterone-derived neurosteroid allopregnanolone, androgen-derived metabolites such as androsterone and 3α-androstanediol can positively modulate GABA-A receptor signaling. These metabolites provide a plausible route through which androgen metabolism might influence affective, stress-related, and reproductive phenotypes; however, direct evidence for this pathway in PMOS remains sparse and does not support clinical endotype assignment (14). PMOS reviews highlight allopregnanolone and propose interacting developmental androgen and later metabolic/hormonal ‘hits’ (14, 95, 96). This supports plausibility, not proof, of an androgen–brain–behavior mechanism.

Small human studies report altered allopregnanolone, GABA-A sensitivity, or cerebrospinal fluid GABA/steroid concentrations, with inconsistent direction and sensitivity to cycle phase, adiposity, treatment, and assay (95, 96). Peripheral levels need not reflect synaptic exposure, and cross-sectional differences cannot separate cause from adaptation; routine endotype testing is premature.

### 7.3 Direct ovarian sympathetic signaling

The ovary receives direct sympathetic innervation. Preclinical PMOS models show increased ovarian signaling and nerve-growth-factor activity, while experimental modulation affects follicles and steroidogenesis (7, 97). This could connect central stress/autonomic regulation with androgen production and ovulatory dysfunction. Human HRV or muscle sympathetic activity does not measure ovarian noradrenergic input, however, and clinical intervention evidence is insufficient.

### 7.4 Brain, reward, gut, and sleep pathways

Insulin, leptin, glucocorticoids, gonadal/neuroactive steroids, sleep loss, and inflammation can affect hypothalamic homeostasis and reward. Distress may change avoidance or reward seeking; restriction increases food salience; OSA adds hypoxia and sympathetic activation. Scarce longitudinal neural data cannot separate predisposition from adaptation.

Human and animal work implicates a gut microbiota–bile acid–interleukin-22 axis and possible gut–brain links (98–100). Diet, metformin, geography, sequencing, handling, and adiposity confound human comparisons. Mouse transfer demonstrates a model mechanism, not human effect size; microbiome tests and supplements are not ready for routine endotyping.

### 7.5 Systems interpretation

A network is more defensible than a single lesion: distress–sleep–fatigue–insulin resistance; stigma–restriction–bingeing–shame; and OSA–hypoxia–autonomic activation–dysglycemia–mood. PMOS biology may enable these loops, while context and treatment shape their strength. Temporally ordered multidomain data are needed to distinguish causes, mediators, and consequences (**Table 2**).

**Table 2:** Direction and evidential strength of proposed psychometabolic pathways.

Proposed direction	Main evidence	What can be concluded	Key uncertainty
PMOS → depression/anxiety	Cross-sectional meta-analyses; symptom persistence in cohorts (20–30, 42–44)	Clinically important association supports repeated assessment.	Direction, ascertainment, shared adversity, and treatment exposure.
Depression/anxiety → metabolic deterioration in PMOS	One retrospective tertiary cohort; adjusted hazard ratio 1.45 for incident metabolic syndrome (101)	Mental-health history may enrich prognosis.	Single setting, residual confounding, exposure and surveillance bias.
PMOS ↔ eating pathology	Meta-analyses, cross-sectional studies, one reverse-direction adolescent cohort (45–56)	Binge/bulimia risk matters before weight treatment.	Few interviews; mediation and temporality largely unknown.
PMOS → OSA/poor sleep	Cross-sectional syntheses and small physiological studies (63–73, 102)	Symptom-based OSA case-finding is justified.	Selection and adiposity; treatment effect on PMOS trajectory unknown.
Stress/autonomic alteration → progression	Stress challenge, hair cortisol, HRV, and microneurography (75–84)	HPA/autonomic differences are plausible signals.	Small, cross-sectional, protocol-sensitive; not diagnostic.
Neuroactive steroid or ovarian sympathetic pathways	Reviews, small human neurosteroid studies, and preclinical ovarian innervation evidence (7, 14, 95–97)	Biologically plausible candidate mechanisms.	Direction, tissue specificity, human temporality, and clinical effect unproven.
Psychological/lifestyle treatment → metabolic improvement	Small heterogeneous PMOS trials and reviews (103–113)	Some outcomes improve; integrated effects are uncertain.	Short follow-up, attrition, weak mediation evidence.
Metabolic treatment → mental/eating improvement	Limited PMOS drug/surgery data; indirect obesity trials (114–130)	Coordinate benefit and harm monitoring.	Indirectness, psychiatric exclusions, withdrawal, confounding.

## 8 Does psychological distress predict metabolic deterioration?

This question requires psychological exposure before metabolic change, adequate follow-up, appropriate confounder control without overadjusting mediators, and preferably replication or intervention.

### 8.1 Direct PMOS evidence

The strongest direct signal is a retrospective cohort of 321 tertiary patients with hyperandrogenic PMOS. Over a median seven years, depression and/or anxiety history predicted incident metabolic syndrome (adjusted hazard ratio (HR) 1.45, 95% CI 1.02–2.06); depression drove the estimate (1.56, 1.10–2.20) (101). This is prognostic, not causal: referral, surveillance, medication, adversity, behavior, misclassification, and severity may confound it, and the setting limits generalization.

Other cohorts confirm later metabolic-syndrome burden (131) or correlate depression trajectories with BMI (43) but do not show that distress causes metabolic change. No adequately powered PMOS cohort has densely repeated mood, eating, sleep, medication, body composition, insulin, and social measures to estimate reciprocal within-person effects; independent anxiety evidence is especially weak.

### 8.2 Indirect longitudinal evidence and causal triangulation

Triangulation integrates methods with different, ideally unrelated biases; agreement strengthens inference and disagreement exposes assumptions (132). General-population evidence contributes but cannot replace PMOS data. In 58,745 participants, a longitudinal meta-analysis found obesity predicted depression (OR 1.55) and depression predicted obesity (OR 1.58) (133). Other studies associate depression with later metabolic syndrome or diabetes (134–138), with residual confounding and heterogeneity.

Mendelian randomization (MR) reduces some confounding only if instrumental-variable assumptions hold. One analysis supported depression liability affecting metabolic syndrome (OR 1.224), not the reverse (139). PMOS-specific results are fragmented: PMOS-to-psychiatric effects were mostly null (140), depression/dysthymia liability may increase PMOS risk (141), and a worry signal was very small (142). Sleep MR explores insomnia and OSA in both directions (143–145). These studies address disease liability, not within-PMOS progression, and remain sensitive to ancestry, phenotype, pleiotropy, instrument strength, overlap, and selection. The longitudinal and causal-inference evidence relevant to the central hypothesis is summarized (**Table 3**).

**Table 3:** Longitudinal and causal-inference evidence relevant to the central hypothesis.

Study/design	Population or exposure	Principal result	Strength	Limitation and role
Lee et al.; retrospective cohort (101)	321 tertiary patients with hyperandrogenic PMOS; depression/anxiety history	Incident metabolic syndrome over median 7 years; adjusted HR 1.45 (95% CI 1.02–2.06)	Temporal outcome and survival model	Single center; historical exposure and residual bias. Direct prediction, not causation.
Greenwood et al.; prospective cohort (43)	163 patients; repeated depression screen over median 5.5 years	Symptoms persisted in many; BMI associated with persistence	Prospective repeated symptom measure	Specialty sample and imprecision. Persistence/prognostic correlation.
Northern Finland Birth Cohort (42)	Questionnaire-defined PMOS symptoms at ages 31 and 46	Distress remained more common; diagnosis awareness linked to anxiety	Population setting and 15-year follow-up	Exposure definition and residual social confounding. Long-term association.
General longitudinal meta-analysis (133)	58,745 participants; obesity and depression	Obesity → depression OR 1.55; depression → obesity OR 1.58	Bidirectional prospective synthesis	Indirect to PMOS. Triangulation only.
Depression–metabolic syndrome MR (139)	Genome-wide association summary data	Depression liability → metabolic syndrome OR 1.224; no reverse effect	Genetic proxy reduces conventional confounding	Pleiotropy and phenotype construction; indirect to within-PMOS progression.
Psychiatric–PMOS MR (140–142)	Primarily European datasets	Mostly null PMOS-to-disorder results; possible depression-to-PMOS and small worry signals	Bidirectional genetic sensitivity analyses	Different estimands, ancestry, power, multiple testing. Mixed disease-liability evidence.
Sleep–PMOS MR (143–145)	European and East Asian datasets	Insomnia/OSA signals vary by phenotype and model	Bidirectional and multivariable analyses	Instrument and adiposity pathways; not treatment effects. Hypothesis generating.

### 8.3 Graded synthesis of current evidence

Evidence is graded: co-occurrence is strong; PMOS-specific temporal prediction is preliminary; causality in either direction is unproven; and no intervention shows that treating distress prevents dysglycemia or diabetes. Clinicians should treat distress without awaiting causal proof, but it should not be described as a metabolic toxin or psychological care as proven diabetes prevention.

## 9 Treatment as a psychometabolic exposure

Treatment may interrupt one pathway while aggravating another; indication, benefit, adverse effects, adherence, access, and priorities must be considered together.

### 9.1 Lifestyle and psychological interventions

PMOS lifestyle trials emphasize weight, hormones, and glucose more than psychological outcomes (103–107). Eight behavioral randomized controlled trials (RCTs; 744 participants) suggested depression improvement with very high heterogeneity (108). A cognitive behavioral therapy (CBT) trial (n=84) reported short-term mood and quality-of-life benefit (109), while a larger CBT-supported lifestyle trial had 63.4% attrition (110). Mind–body and diet trials remain small and heterogeneous (111–113).

Use functional goals—regular meals, enjoyable movement, stable wake time, less bingeing, or medication adherence—and do not make weight the sole engagement measure. Active eating disorders require nutritional adequacy and specialist care before aggressive restriction.

### 9.2 Metformin, combined oral contraceptive pills, and antiandrogens

Metformin supports selected metabolic/cycle indications; combined oral contraceptive pills (COCPs) are widely used for cycle and androgenic symptoms; and antiandrogens address hirsutism/acne. Their benefit–harm profiles differ (114–116), so indication, contraindications, reproductive plans, and preference govern choice.

COCP trials rarely prioritize mental health. A 2025 review describes hormonal, immune, and neural pathways but finds depression effects heterogeneous and entangled with symptom improvement, fertility concerns, baseline vulnerability, and formulation (130). COCPs are neither antidepressants nor uniformly depressogenic. Record baseline mood and previous hormone-related change; after starting or switching, reassess temporal symptoms, adherence, bleeding, and valued benefit. Important deterioration warrants diagnostic review and shared modification, not automatic causal attribution.

Metformin gastrointestinal effects may disrupt eating/adherence; antiandrogens require pregnancy prevention and monitoring. Measure mental and reproductive outcomes rather than inferring one from the other.

### 9.3 Anti-obesity medication and glucagon-like peptide-1 receptor agonists

The 2023 guideline review found 11 PMOS anti-obesity-drug trials (117); later glucagon-like peptide-1 receptor agonist (GLP-1 RA) syntheses report weight and some metabolic improvement (118, 119). Liraglutide trials show weight/ectopic-fat effects and one open-label trial examined semaglutide plus metformin (120–122). Reproductive, mood, eating-safety, diversity, and withdrawal data remain inadequate.

General obesity trials report reduced appetite and loss-of-control ratings with semaglutide (123, 124), but are indirect. About two-thirds of prior weight loss returned within one year after STEP 1 withdrawal (125). RCT meta-analyses have not detected excess psychiatric/suicidal outcomes (126, 127), but rare events and high-risk groups remain uncertain. Screen, monitor, plan maintenance/pregnancy, and do not use a GLP-1 RA to treat an eating disorder.

### 9.4 Metabolic surgery

Metabolic surgery can improve PMOS features in eligible patients, although evidence is mainly uncontrolled (128). General-population evidence suggests early mood/binge benefit but later alcohol, self-harm, and suicide risk (129). Assess psychiatric stability, eating, substances, expectations, support, and lifelong nutrition before and after surgery without making review an inappropriate barrier.

### 9.5 Psychiatric pharmacotherapy, antipsychotics, and valproate

Untreated depression, bipolar disorder, psychosis, and severe anxiety carry major risk; metabolic factors should refine, not displace, effective treatment. Antidepressants differ in cardiometabolic effects, observational weight/diabetes associations remain confounded, and short diabetes trials provide limited joint mood–glycemic evidence (146–150). Prior response, safety, pregnancy plans, eating, sleep, interactions, and access guide selection.

Antipsychotics may increase weight, lipids, and glycemia; prolactin can alter menstruation, with agent/dose differences (151). A 2025 pharmacovigilance study found disproportionate PMOS reports for olanzapine and quetiapine, but spontaneous reports cannot establish incidence or causality (152). Coordinate baseline/follow-up weight or waist, blood pressure, glycemia, lipids, cycles, and indicated prolactin monitoring.

Valproate is associated with weight gain, menstrual irregularity, hyperandrogenic features, and PCOS-like presentations. A 2023 network meta-analysis ranked valproate highest among four anticonvulsant mood stabilizers for facilitating PCOS, with heterogeneity and indication confounding (153). New symptoms require temporal assessment and exclusion of alternatives; medication-associated change is not automatically primary PMOS. Never stop psychotropics abruptly; coordinate any monitored alternative.

## 10 Integrated clinical formulation pathway

A six-step pathway moves from priorities and safety to assessment, shared formulation, treatment, and monitoring. Care-intensity tiers are nested within Step 5, eliminating the former parallel scheme. This supports reasoning but is neither a new diagnostic criterion nor a validated guideline (**Figure 2**).

**Figure 2:**
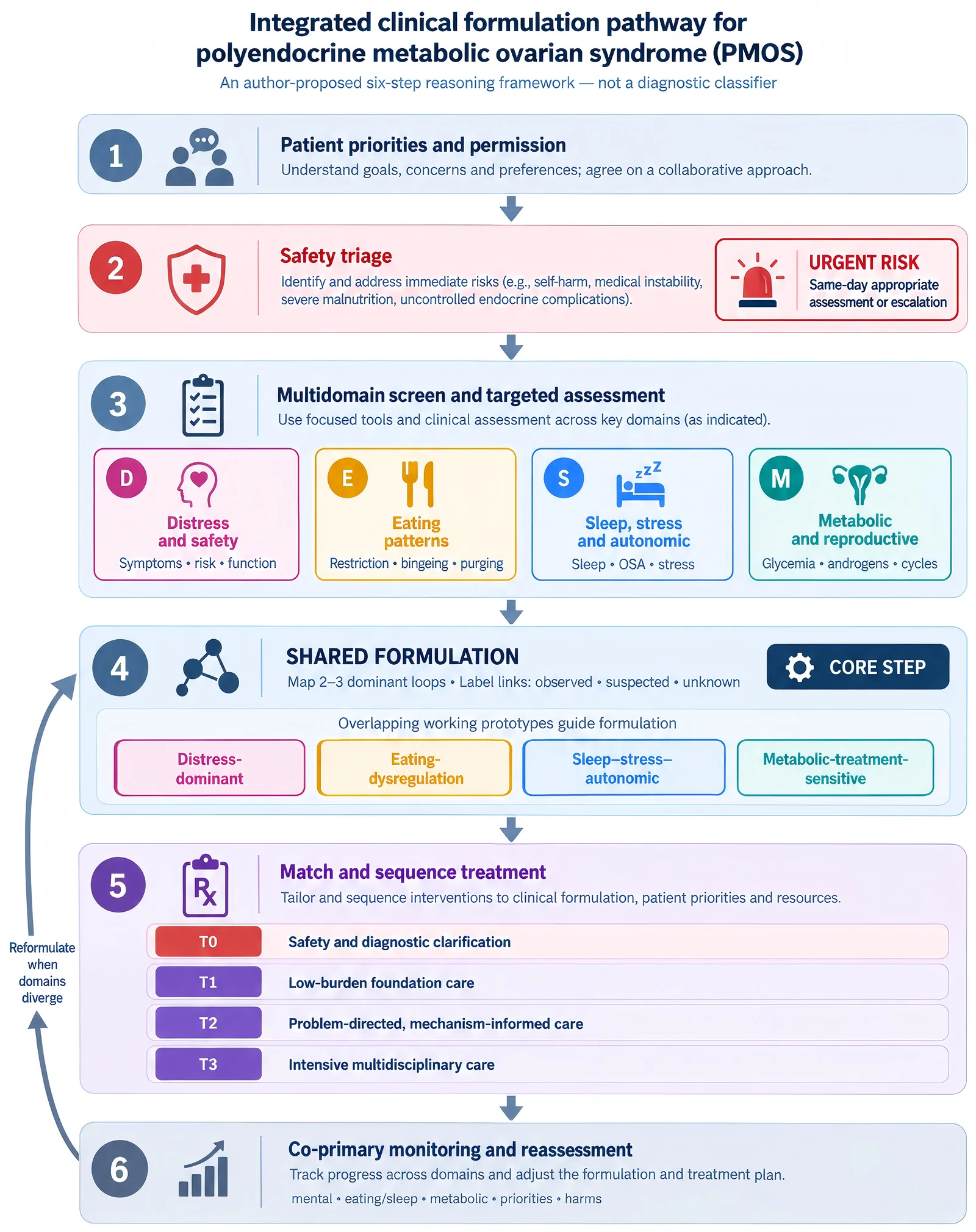
Integrated clinical formulation pathway for polyendocrine metabolic ovarian syndrome (PMOS). The six-step pathway moves from patient priorities and safety triage through multidomain assessment to shared formulation as the central clinical step. Four care-intensity tiers are nested within treatment matching, and co-primary psychological and metabolic–reproductive monitoring triggers reformulation when domains diverge. The dark outline identifies Step 4 as the clinical core. Colors are concept-specific: red denotes safety, purple denotes treatment matching, and gray-blue denotes monitoring and reassessment. Domain colors are carried over from **Figure 1** only when they represent the same domain. This author-proposed pathway supports clinical reasoning but is not a validated diagnostic or treatment guideline.

### 10.1 Step 1: ask permission and define priorities

Start with goals—cycles, fertility, hirsutism, mood, energy, sleep, eating, or metabolic prevention—and ask permission before weight discussion or measurement. Record gender identity and preferred language where relevant; PMOS affects some people who do not identify as women, although most evidence studies women.

### 10.2 Step 2: triage safety

Suicidality, severe self-neglect, dangerous purging, syncope, rapid unexplained loss, pregnancy urgency, metabolic decompensation, or suspected severe OSA supersedes routine planning. Define same-day escalation and retain responsibility until handover.

### 10.3 Step 3: screen and complete a targeted assessment

At diagnosis and meaningful change, assess mood/safety, indicated bipolar or psychosis history, eating, sleep/OSA, medications/substances, activity, stigma, food security, reproductive goals, and metabolic risk. Tools improve consistency (**Table 4**) but do not diagnose. Establish timing: Did mood precede medication or weight change? Does bingeing follow restriction or sleep loss? Is fatigue multifactorial?

**Table 4:** Pragmatic multidomain assessment for routine PMOS care.

Domain	Brief signal or tool	Meaning	Action and caveat
Depression and safety	9-item Patient Health Questionnaire (PHQ-9); interview (154)	Symptom burden; item 9 is a safety signal	Assess duration, impairment, bipolar history, self-harm, and suicide risk. Escalate urgent risk; a score is not a diagnosis.
Anxiety	7-item Generalized Anxiety Disorder scale (GAD-7) or Hospital Anxiety and Depression Scale (HADS) (155, 156)	General anxiety burden	Assess panic, obsessive-compulsive, trauma, social, or illness anxiety as indicated.
Eating pathology	Direct questions plus 7-item Binge-Eating Disorder Screener (BEDS-7) (157)	Possible loss of control or binge-eating disorder	Ask separately about restriction, purging, laxatives, and compensatory exercise; arrange diagnostic assessment.
Body image/stigma	Appearance distress, avoidance, PCOSQ or PCOSQOL (33, 34)	Symptoms or care experience may drive distress	Address manifestations and healthcare environment; BMI is not a stigma measure.
Sleep and OSA	Duration/timing, Pittsburgh Sleep Quality Index (PSQI) (158); snoring, apnea, sleepiness, blood pressure; STOP (Snoring, Tiredness, Observed apnea, and high blood pressure) questionnaire where locally appropriate (159)	Poor sleep or need for diagnostic OSA evaluation	Clarify schedule, substances, caregiving, restless legs, and mood; questionnaires do not diagnose OSA and may underperform in younger women.
Stress/context	Life events, trauma, discrimination, food security, shift work, caregiving	Potential common causes or modifiers	Link to social and psychological support; do not translate adversity into an unqualified cortisol explanation.
Activity	History; International Physical Activity Questionnaire (IPAQ) for standardized research use (160)	Movement and sedentary pattern	Explore function, pain, safety, preference, and access; self-report is not fitness testing.
Metabolic status	Purposeful weight/waist trajectory, blood pressure, lipids, glycemia, guideline-directed oral glucose tolerance test (2)	Current risk and change	Interpret by age, pregnancy, medication, and comorbidity; HOMA-IR has no universal clinical cutoff.
Treatment exposure	Medication/supplement reconciliation, contraception, fertility, anti-obesity and psychotropic drugs	Benefit, adverse effect, interaction, or indication bias	Record start/stop timing relative to mood, eating, sleep, cycles, and metabolic change; temporal coincidence is not proof.

Confirm PMOS and indicated mimics. Assess blood pressure, lipids, glycemia, and purposeful weight/waist trajectory; an oral glucose tolerance test can outperform fasting glucose (2). Insulin assays and HOMA-IR lack universal clinical thresholds (2).

### 10.4 Step 4: construct a shared formulation—the core clinical contribution

With the patient, convert parallel findings into an actionable map of two or three dominant loops: OSA → fatigue → inactivity → dysglycemia; stigma → restriction → bingeing → shame; depression → irregular sleep/medication → deterioration; or antipsychotic exposure → appetite/weight → insulin risk/distress. Label links observed, suspected, or unknown. Formulation—not prototype assignment—sets priorities.

Shared formulation avoids determinism, exposes trade-offs, and gives disciplines one map; revise it whenever domains diverge.

### 10.5 Step 5: match and sequence treatment using nested care-intensity tiers

Intensity follows need and interaction, not BMI. These nested tiers can be concurrent and bidirectional:

• Tier 0—safety/diagnostic clarification: address acute psychiatric, eating, metabolic, pregnancy, or sleep risk; confirm diagnoses and mimics.
• Tier 1—foundation: education, respectful language, regular sleep/meals, feasible movement, symptom care, medication review, and barrier support.
• Tier 2—problem-directed: indicated psychotherapy, eating-disorder nutrition/treatment, sleep care, reproductive–endocrine, metabolic, fertility, or anti-obesity therapy.
• Tier 3—intensive multidisciplinary: coordinate specialties for severe or reinforcing disease with purposeful, closed-loop referral.

Predefine stopping/switching for worsening bingeing or suicidality, intolerable effects, incompatible pregnancy plans, inadequate benefit, or unaffordable continuation (**Table 5**).

**Table 5:** Treatment matrix within the shared formulation.

Intervention	Best-fit problem	Mental/eating considerations	Metabolic/reproductive considerations	Evidence boundary/monitoring
Weight-inclusive lifestyle support	All prototypes when feasible	Flexible goals; screen before restriction; monitor burden and bingeing	Supports movement, sleep, glycemia, and cardiometabolic health	PMOS trials heterogeneous; assess function and harms, not weight alone (103–107).
CBT or disorder-specific therapy	Distress, restriction–binge loop, insomnia when cognitive behavioral therapy for insomnia is available	Assess diagnosis/safety; use eating-disorder-specific care when indicated	Indirect benefit through sleep, self-care, or adherence is plausible	Small/short PMOS trials with heterogeneity and attrition (108–112).
Dietetic care	Eating dysregulation, food insecurity, metabolic risk	Regular nourishment before aggressive deficit; avoid moralized rules	Individualize adequacy, affordability, culture, and pregnancy needs	No single diet has established psychometabolic superiority (103–107, 113).
OSA/insomnia treatment	Sleep–stress–autonomic prototype	Monitor energy, mood, insomnia, and treatment burden	May improve sleep physiology; PMOS metabolic outcome trials limited	Confirm OSA objectively; symptom change does not prove metabolic cure (63–73, 102).
Metformin	Dysglycemia/insulin risk; selected cycle indications	Gastrointestinal effects may disrupt eating/adherence	Evidence-based role varies by phenotype and goal	Monitor tolerance, renal context, vitamin B12 as indicated, and reproductive plan (2, 114, 115).
COCP/antiandrogen	Cycle control, acne, hirsutism	Document baseline mood and hormone-related history; reassess temporal change	Consider vascular risk, contraception, and pregnancy prevention	Depression data sparse/heterogeneous; do not assume class mood effect (114–116, 130).
GLP-1 RA/other anti-obesity drug	Eligible metabolic-treatment-sensitive profile	Assess eating disorder, mood, nausea, suicidality, cost; not treatment for an eating disorder	Improves weight and some metabolic measures; plan pregnancy/maintenance	PMOS data limited; withdrawal regain common; rare-event uncertainty (117–127).
Antidepressant	Indicated depressive/anxiety disorder	Prior response, safety, sleep, eating symptoms, withdrawal	Agents differ in cardiometabolic effects	Individualized monitoring; observational associations are not fully causal (146–150).
Antipsychotic or valproate	Indicated psychosis/bipolar disorder/epilepsy	Do not stop abruptly; preserve psychiatric stability	Monitor weight/waist, blood pressure, glycemia, lipids, cycles; prolactin as indicated	Agent-specific risk; valproate and some antipsychotics linked to PCOS-like features (151–153).
Metabolic surgery	Eligible severe obesity/metabolic disease	Assess eating, substance use, expectations, psychiatric stability; follow long term	Can markedly improve weight-related and PMOS outcomes	PMOS data mostly uncontrolled; later alcohol/self-harm risks require monitoring (128, 129).

### 10.6 Step 6: monitor benefit and harm as co-primary outcomes

Track one mental, one eating/sleep, one metabolic, one patient-priority outcome, and harms. Reassess after change and by risk; discordant outcomes trigger reformulation, not automatic escalation.

### 10.7 Implementation guardrails

Screening without response capacity can harm. Define review, escalation, ownership, and handover; keep measurement proportionate. Weight measurement needs a purpose and may be blind or declined when nonessential. Do not praise loss before understanding its cause or attribute gain automatically to behavior.

Name trade-offs: an effective psychiatric drug may outweigh metabolic risk; a GLP-1 RA may improve glycemia while disrupting meals; fertility may alter sequencing. Evaluate urgent access, referral completion, joint outcomes, adverse events, burden, stigma, and inequity—not only referrals or weight (2, 13, 38–41).

Weight-inclusive and eating-disorder-aware language is a clinical safety intervention. Ask permission before discussing weight or recent weight change (for example, ‘Would it be acceptable to discuss how weight or recent weight change may relate to your health today?’). State explicitly that an eating disorder can occur at any body size, and ask about loss-of-control eating, severe restriction, vomiting, laxative use, or compensatory exercise. Ask which outcome matters most over the next three months—mood, sleep, eating regularity, cycles, fertility, energy, symptoms, or a metabolic measure. Avoid telling a person that they ‘just need to lose weight’, labeling them ‘noncompliant’ before feasibility and adverse effects are assessed, assuming that BMI proves or excludes an eating disorder, or promising that one diet, biomarker, supplement, or drug will reverse PMOS.

## 11 Life-course transitions

In adolescence, diagnostic uncertainty overlaps with pubertal irregularity and acne. Screen for bullying, body-image distress, eating disorders, sleep schedule, family dynamics, and school function while following adolescent-specific diagnostic recommendations (11, 28, 29, 54). Avoid making future infertility the central educational message.

During fertility treatment, uncertainty, repeated procedures, weight eligibility rules, and medication schedules may intensify distress or disordered eating. Preconception care should include coordinated psychiatric-medication review without abrupt withdrawal, eating-disorder and OSA assessment, glycemic evaluation, and realistic counseling. Pregnancy and postpartum care require surveillance for dysglycemia, hypertensive complications, sleep disruption, and depression; prior psychiatric history remains a major contextual risk, and PMOS-specific postpartum estimates should not be treated as causal (30).

At midlife and menopause, menstrual markers become less informative while cumulative adiposity, dysglycemia, blood pressure, OSA, and cardiovascular risk become more salient. Mood and sleep symptoms may overlap with menopausal transition, caregiving, and chronic disease. Historical PMOS should prompt continued risk assessment without attributing every new symptom to the syndrome (10, 74, 131).

## 12 Validation roadmap: how the endotype could be confirmed or rejected

The proposed construct must be allowed to fail. Validation begins with a shared minimum dataset and an explicit estimand: discovery of co-variation, prediction of trajectory, explanation of a causal pathway, or identification of treatment-response heterogeneity. A cross-sectional correlation or algorithmic cluster can support discovery but cannot establish an endotype.

### 12.1 Construct and measurement validation

Prospective cohorts should measure psychological symptoms and diagnoses, eating behavior, objective and reported sleep, stress/context, medication exposure, reproductive features, adiposity, and metabolic outcomes repeatedly at intervals capable of resolving sequence. Measurement invariance should be tested across age, language, ancestry, body size, and life stage. Objective sleep testing, pharmacy data, menstrual tracking, body composition, and oral glucose tolerance can complement validated patient-reported outcomes. Attrition and missingness must be modeled because distress and socioeconomic constraint may predict loss to follow-up.

### 12.2 Discovery, stability, and transition

Latent-class, latent-profile, network, or multi-omic clustering may discover candidate patterns, but internal separation is insufficient. Stability should be tested across algorithms, preprocessing decisions, diagnostic criteria, ancestry, and settings. Bootstrap or split-sample stability, external replication, and comparison with simple baselines are essential. A pattern that merely reconstructs BMI or Rotterdam phenotype is useful stratification, not a new mechanism. Latent-transition and dynamic models may be more realistic than permanent classes and can test movement among prototypes.

### 12.3 Temporal, prognostic, and causal validation

Cross-lagged, random-intercept, growth-curve, joint, or dynamic structural-equation models can test whether within-person change in one domain precedes another while separating stable between-person differences. Prognostic models should report calibration, discrimination, incremental value, missing-data handling, and external validation beyond NIH/Rotterdam phenotype, adiposity, baseline metabolic status, medications, and social determinants. Causal analyses should prespecify directed acyclic graphs, separate confounders from mediators, address time-varying confounding, and use negative controls, target-trial emulation, or genetically informed designs where assumptions are credible. Triangulation requires concordance across methods with distinct biases, not repeated use of the same cross-sectional dataset (132).

### 12.4 Treatment-response validation

The strongest clinical validation would be replicated treatment-effect heterogeneity: a prespecified endotype measure modifies response to a mechanism-matched intervention. Trials should capture mental and metabolic co-primary or key secondary outcomes, adverse eating and psychiatric events, burden, and maintenance after withdrawal. Factorial or sequential multiple-assignment designs can test whether treating sleep, eating pathology, or depression changes response to metabolic therapy. Mediation requires temporal ordering and sensitivity analysis; improvement within a subgroup or significance in only one arm is not evidence of interaction. Multiplicity control and external replication are mandatory.

### 12.5 Equity and clinical utility

Validation samples should include adolescents, midlife adults, gender-diverse people with ovaries where appropriate, racial and ethnic groups underrepresented in existing cohorts, rural populations, and people across body sizes and socioeconomic positions. Measures require cultural and linguistic validation. Decision-curve analysis, burden, cost, access, stigma, and patient-defined function should accompany biomarker performance. A complex assay must add value beyond a brief clinical formulation before implementation.

### 12.6 Minimum dataset for psychometabolic-endotype research

Repeated core domains should include diagnostic criteria and age at diagnosis; menstrual/ovulatory status; clinical and biochemical hyperandrogenism; reproductive stage and goals; anthropometry and weight trajectory; blood pressure; glycemia (oral glucose tolerance where feasible); lipids; medication start/stop dates; depression/anxiety symptoms and diagnoses; suicidality; bingeing, purging, restriction, and loss of control; sleep duration/timing, insomnia, sleepiness and OSA testing; activity/sedentary time; smoking, alcohol and substance use; quality of life; and a patient-priority outcome.

Context and modifiers should include race and ethnicity as social and ancestry variables rather than biological shortcuts; gender identity; education, income, employment and shift work; food security; caregiving; trauma and life events; weight stigma and healthcare discrimination; geography and access; fertility treatment; pregnancy/postpartum; menopause; and relevant comorbidity.

Design requirements include timestamped repeated measures; a preregistered causal or prognostic question; explicit confounder and mediator roles; missing-data and attrition analyses; external validation; treatment exposure and adherence; adverse psychiatric and eating outcomes; patient involvement; a data dictionary and code; and calibrated prediction rather than cluster labels alone.

## 13 Discussion and conclusions

Psychological distress, eating dysregulation, sleep disturbance, and metabolic dysfunction are clinically important and often intertwined in PMOS. Association evidence is substantial; PMOS-specific temporal evidence is limited; causal evidence is inconsistent and indirect; and treatment studies seldom measure the full system. The psychometabolic construct should therefore remain candidate and testable.

Its immediate utility is organizational rather than classificatory. It directs attention beyond weight, places safety before restriction, highlights OSA and eating disorders, integrates the metabolic consequences of psychiatric treatment, and supports joint monitoring. The four prototypes are shared-formulation tools, not diagnoses. A true endotype must demonstrate replicated longitudinal coupling and differential clinical value beyond existing phenotypes and ordinary risk factors. Until then, integrated and non-stigmatizing care is justified by patient need—not by a claim of proven psychometabolic causality.

## Supplementary material

Additional file 1 Search strategy and study-selection framework (Word document): exact database strings, record yields, eligibility and prioritization criteria, study-selection process, and domain-selection rationale.
