# Supplementary material for: A proposed psychometabolic endotype of polyendocrine metabolic ovarian syndrome: bidirectional interplay between psychological distress, eating dysfunction, sleep disruption, chronic stress, and metabolic deterioration
Source: Front Endocrinol (Lausanne). 2026 Oct 7;17:1978249. doi: 10.3389/fendo.2026.1978249 (PMC13684126; doi:10.3389/fendo.2026.1978249)
Supplement: Supplementary file 1 [file Table1.docx]

**Additional file 1. Search strategy and study-selection framework**

# **Search overview**

Database: PubMed/MEDLINE. Coverage: database inception through 15 September 2026. Language: English. Seven structured topic blocks yielded 1,278 records before cross-search deduplication and 1,009 unique PubMed identifiers after PubMed identifier (PMID)-based deduplication. Of these, 1,008 article records were retrievable and constituted the title/abstract screening set; one identifier did not return a PubmedArticle record at export. Reference chaining and professional-society or publisher records identified relevant guidelines and source reports outside the topic-block retrieval. The final manuscript cites 160 sources. All record counts reflect searches executed on 15 September 2026 and are reported as historical search yields; subsequent changes in PubMed indexing do not alter these recorded counts.

# **Table S1. Exact search strings and yields**

| **No.** | **Domain** | **Exact PubMed/MEDLINE search string** | **Records** |
| --- | --- | --- | --- |
| 1 | Mental health and lived experience | ("polycystic ovary syndrome"[Title/Abstract] OR "polycystic ovarian syndrome"[Title/Abstract] OR "polyendocrine metabolic ovarian syndrome"[Title/Abstract]) AND (depress*[Title/Abstract] OR anxi*[Title/Abstract] OR distress*[Title/Abstract] OR "body image"[Title/Abstract] OR stigma*[Title/Abstract] OR "quality of life"[Title/Abstract]) AND ("systematic review"[Title/Abstract] OR meta-analysis[Title/Abstract] OR longitudinal[Title/Abstract] OR prospective[Title/Abstract] OR cohort[Title/Abstract] OR guideline[Title/Abstract] OR "randomized controlled trial"[Title/Abstract]) AND ("1900/01/01"[Date - Publication] : "2026/09/15"[Date - Publication]) AND english[Language] | 408 |
| 2 | Eating behavior | ("polycystic ovary syndrome"[Title/Abstract] OR "polycystic ovarian syndrome"[Title/Abstract] OR "polyendocrine metabolic ovarian syndrome"[Title/Abstract]) AND ("eating disorder"[Title/Abstract] OR "disordered eating"[Title/Abstract] OR binge*[Title/Abstract] OR bulimi*[Title/Abstract] OR anorexi*[Title/Abstract] OR "emotional eating"[Title/Abstract] OR "dietary restraint"[Title/Abstract] OR "food reward"[Title/Abstract] OR palatab*[Title/Abstract]) AND ("1900/01/01"[Date - Publication] : "2026/09/15"[Date - Publication]) AND english[Language] | 148 |
| 3 | Sleep and circadian function | ("polycystic ovary syndrome"[Title/Abstract] OR "polycystic ovarian syndrome"[Title/Abstract] OR "polyendocrine metabolic ovarian syndrome"[Title/Abstract]) AND (sleep[Title/Abstract] OR insomnia[Title/Abstract] OR "sleep apnea"[Title/Abstract] OR circadian[Title/Abstract] OR chronotype[Title/Abstract]) AND ("systematic review"[Title/Abstract] OR meta-analysis[Title/Abstract] OR cohort[Title/Abstract] OR longitudinal[Title/Abstract] OR prospective[Title/Abstract] OR "case-control"[Title/Abstract] OR polysomnograph*[Title/Abstract] OR clamp[Title/Abstract] OR "Mendelian randomization"[Title/Abstract]) AND ("1900/01/01"[Date - Publication] : "2026/09/15"[Date - Publication]) AND english[Language] | 130 |
| 4 | Stress autonomic and neuroendocrine mechanisms | ("polycystic ovary syndrome"[Title/Abstract] OR "polycystic ovarian syndrome"[Title/Abstract] OR "polyendocrine metabolic ovarian syndrome"[Title/Abstract]) AND (cortisol[Title] OR "HPA axis"[Title] OR autonomic[Title] OR sympathetic[Title] OR neurosteroid*[Title] OR allopregnanolone[Title] OR GABA[Title] OR neuroimaging[Title] OR "brain activation"[Title]) AND ("1900/01/01"[Date - Publication] : "2026/09/15"[Date - Publication]) AND english[Language] | 125 |
| 5 | Longitudinal and causal evidence | ("polycystic ovary syndrome"[Title/Abstract] OR "polycystic ovarian syndrome"[Title/Abstract] OR "polyendocrine metabolic ovarian syndrome"[Title/Abstract]) AND (depress*[Title/Abstract] OR anxi*[Title/Abstract] OR distress*[Title/Abstract] OR "eating disorder"[Title/Abstract] OR binge*[Title/Abstract] OR sleep[Title/Abstract] OR insomnia[Title/Abstract]) AND (longitudinal[Title/Abstract] OR prospective[Title/Abstract] OR trajectory[Title/Abstract] OR "Mendelian randomization"[Title/Abstract] OR "causal inference"[Title/Abstract] OR mediation[Title/Abstract]) AND ("1900/01/01"[Date - Publication] : "2026/09/15"[Date - Publication]) AND english[Language] | 150 |
| 6 | Treatment exposures | ("polycystic ovary syndrome"[Title/Abstract] OR "polycystic ovarian syndrome"[Title/Abstract] OR "polyendocrine metabolic ovarian syndrome"[Title/Abstract]) AND (psychotherap*[Title/Abstract] OR "cognitive behavioral"[Title/Abstract] OR contracept*[Title/Abstract] OR "GLP-1"[Title/Abstract] OR semaglutide[Title/Abstract] OR liraglutide[Title/Abstract] OR bariatric[Title/Abstract] OR "metabolic surgery"[Title/Abstract] OR antidepress*[Title/Abstract] OR antipsychotic*[Title/Abstract] OR valpro*[Title/Abstract]) AND (depress*[Title/Abstract] OR anxi*[Title/Abstract] OR mood[Title/Abstract] OR eating[Title/Abstract] OR sleep[Title/Abstract] OR "quality of life"[Title/Abstract]) AND ("1900/01/01"[Date - Publication] : "2026/09/15"[Date - Publication]) AND english[Language] | 260 |
| 7 | Phenotypes endotypes and clusters | ("polycystic ovary syndrome"[Title/Abstract] OR "polycystic ovarian syndrome"[Title/Abstract] OR "polyendocrine metabolic ovarian syndrome"[Title/Abstract]) AND (endotype*[Title] OR subtype*[Title] OR cluster*[Title] OR "latent class"[Title] OR "phenotypic clustering"[Title]) AND ("1900/01/01"[Date - Publication] : "2026/09/15"[Date - Publication]) AND english[Language] | 57 |

# **Table S2. Eligibility and prioritization**

| **Element** | **Approach used for the targeted narrative search** |
| --- | --- |
| Population | People with PMOS/PCOS across adolescence, reproductive years, pregnancy/postpartum, and later life. General-population evidence was eligible only to address a direction, mechanism, or treatment-safety question not adequately studied in PMOS and was labeled indirect. |
| Core domains | Depression/anxiety; body image and weight stigma; eating pathology; sleep/OSA/circadian function; stress, HPA, and autonomic regulation; metabolic/reproductive outcomes; treatment exposure; conventional phenotypes and data-driven clusters. |
| Eligible evidence | International guidelines and consensus statements; systematic reviews/meta-analyses; longitudinal cohorts; Mendelian-randomization or other causal-inference studies; randomized and informative nonrandomized intervention studies; human mechanistic studies; selected preclinical evidence for ovarian sympathetic or other mechanistic plausibility. |
| Design priority | Guideline-level evidence and systematic synthesis first; longitudinal/causal studies for direction; trials for treatment effects; mechanistic human studies for biological plausibility. Landmark studies were retained when they clarified a key design or mechanism. |
| Excluded or not prioritized | Non-English records; reports without a retrievable full article; isolated case reports; sources unrelated to the review question; duplicative reviews that added no material evidence. Cross-sectional and preclinical studies were not used to claim causation. |
| Selection rationale | Relevance to one of five evidential roles: burden/association, temporality, causal inference, intervention, or implementation. When reviews overlapped, the most recent or methodologically informative synthesis was prioritized. |

# **Study-selection process and limitations**

- Search results were merged and deduplicated by PubMed identifier; 1,009 unique identifiers remained and 1,008 article records were retrievable for screening.
- The authors conducted a single-stage title-and-abstract relevance screen of the 1,008 retrievable records to identify potentially informative reports for full-text checking and citation. Uncertain records were resolved by discussion within the review team. No independent duplicate screening was claimed.
- Potentially relevant sources were assessed for population, domain, design, evidential role, and directness to PMOS. All eligibility, citation, and interpretive decisions remained the authors' responsibility.
- Because this was a targeted narrative review, no PRISMA flow diagram, formal risk-of-bias score, exhaustive full-text exclusion log, or meta-analysis was produced. Record counts describe the reproducible search set, not a systematic-review claim.
- Cross-sectional, genetic, general-population, and preclinical evidence is labeled according to its inferential boundary in the manuscript tables and narrative.

# **Domain-selection rationale**

The core domains were selected for the targeted narrative synthesis because they are repeatedly identified in international PMOS guidance or syntheses, can plausibly alter both care behavior and metabolic physiology, and provide measurable, potentially modifiable targets. Bipolar disorder, psychotic disorders, attention-deficit/hyperactivity disorder, and autism spectrum conditions were not treated as core domains because PMOS-specific longitudinal and mechanistic evidence is less developed or heterogeneous. They remain important comorbidities, differential diagnoses, and treatment-exposure contexts and are addressed when clinically relevant.
